# Characterization and Use in Wheat Breeding of Leaf Rust Resistance Genes from Durable Varieties

**DOI:** 10.3390/biology10111168

**Published:** 2021-11-12

**Authors:** María José Diéguez, Micaela López, Emiliano Altieri, María Fernanda Pergolesi, Marisol Alicia Dabove, Alba Romina Cuyeu, Nadia Justus, Mariana Kandus, Lorena Ingala, Francisco Sacco

**Affiliations:** 1 Instituto de Genética “Ewald A. Favret”, Centro de Investigación en Ciencias Veterinarias y Agronómicas, Instituto Nacional de Tecnología Agropecuaria CC25 (1712), Castelar B1712, Argentina; lopez.micaela@inta.gob.ar (M.L.); emilianoaltieri@hotmail.com (E.A.); pergolesi.maria@inta.gob.ar (M.F.P.); dabove.marisol@inta.gob.ar (M.A.D.); cuyeu.alba@inta.gob.ar (A.R.C.); justus.nadia@inta.gob.ar (N.J.); kandus.mariana@inta.gob.ar (M.K.); ingala.lorena@inta.gob.ar (L.I.); sacco.francisco@inta.gob.ar (F.S.); 2 Instituto de Recursos Biológicos, Centro de Investigación de Recursos Naturales, Instituto Nacional de Tecnología Agropecuaria CC25 (1712), Castelar B1712, Argentina

**Keywords:** wheat leaf rust, durable resistance, adult plant resistance genes, marker-assisted selection, gene introgression, fine mapping

## Abstract

**Simple Summary:**

Wheat leaf rust is one of the most significant diseases worldwide, incited by a parasitic fungus which infects leaves, affecting grain yield. This pathogen is spread by the wind over large areas through microscopic spores. This huge number of spores favors the selection of virulent forms; therefore, there is a continuous need for new resistance genes to control this disease without fungicides. These resistant genes are naturally found in resistant wheat varieties and can be introduced by standard crosses. In this work, seven resistant genes were introduced into several commercial susceptible varieties. The selection of resistance genes was assisted by DNA markers that are close to these genes on the chromosome. Additionally, the selection of desirable traits from the commercial variety was also assisted by DNA markers to accelerate the process. In field testing, the varieties developed here were resistant to leaf rust, and suitable for commercial use.

**Abstract:**

Leaf rust is one of the most significant diseases of wheat worldwide. In Argentina, it is one of the main reasons for variety replacement that becomes susceptible after large-scale use. Some varieties showed durable resistance to this disease, including Buck Manantial and Sinvalocho MA. RILs (Recombinant Inbred Lines) were developed for each of these varieties and used in genetics studies to identify components of resistance, both in greenhouse inoculations using leaf rust races, and in field evaluations under natural population infections. In Buck Manantial, the APR gene *LrBMP1* was associated with resistance in field tests. In crosses involving Sinvalocho MA, four genes were previously identified and associated with resistance in field testing: APR (Adult Plant Resistance) gene *LrSV1*, the APR genetic system *LrSV2* + *LrcSV2* and the ASR (All Stage Resistance) gene *LrG6*. Using backcrosses, *LrBMP1* was introgressed in four commercial susceptible varieties and *LrSV1*, *LrSV2* + *LrcSV2* and *LrG6* were simultaneously introgressed in three susceptible commercial varieties. The use of molecular markers for recurrent parent background selection allowed us to select resistant lines with more than 80% similarity to commercial varieties. Additionally, progress towards positional cloning of the genetic system *LrSV2* + *LrcSV2* for leaf rust APR is reported.

## 1. Introduction

World wheat production in the last five years was over 700 million tons. This cereal has become one of the most important sources of food for humans, providing energy, fiber and essential nutrients, and to a lesser extent for animal nutrition. In Argentina, wheat annual production was around 15–20 million tons (last five years), reaching a value of more than USD 3000 million per year, according to current wheat prices on the world market. An additional value of wheat is that this crop is usually grown associated in rotation to soybean, playing an important role in soil structure conservation. 

The main objectives in wheat breeding programs are high yield, good quality and reducing losses due to environmental stress and biotic factors, although it is difficult to combine them simultaneously. Concerning wheat fungal diseases, rusts cause important yield losses. The three wheat rusts: stem rust, leaf rust and yellow rust, are incited by different species of *Puccinia*, impacting on wheat yield according to environmental conditions and resistant genes present in cultivated varieties. Leaf rust, caused by the biotrophic fungus *Puccinia triticina*, is probably the most important disease of wheat on a worldwide historical basis due to its more frequent and widespread occurrence [1]. In Argentina, it causes annual yield losses of about 5–10% [2,3,4,5]. Most of the new wheat commercial varieties are selected for resistance to current leaf rust populations. However, they frequently become susceptible when widely grown over the years because of the occurrence and selection of new virulent strains, as was the case of many varieties, including Klein Don Enrique, Klein Cacique, ProINTA Gaucho and DM Algarrobo (https://www.argentina.gob.ar/inase/red-de-ensayos-comparativos-de-variedades-de-trigo/grupo-de-calidad-de-trigo-pan (accessed on 30 August 2021), [1]. Changes in leaf rust pathogen populations during 1996–2003 affected 10 cultivars and caused estimated yield losses of USD 172 million in Argentina, Brazil, Chile, Paraguay and Uruguay [6]. The costs of annual fungicide applications from 1999 to 2003 were estimated at more than USD 50 million and potential yield losses in areas with favorable weather conditions for leaf rust development in the Southern Cone of South America can exceed 50% if fungicides are not applied [1].

The use of resistance genes can reduce or eliminate fungicide applications. The genetic bases of rust resistance have been established by the pioneering work of Harold Flor on flax rust, who proposed the gene for gene theory for the relationship between host and pathogen and the epistatic effects among them [7]. This kind of interaction was confirmed for wheat leaf rust [8]. More than 80 leaf rust resistance genes have been described, not only from wheat, but also from wheat-related species, that have been introduced into the wheat genome by different methodologies [9,10].

Leaf rust resistance related to single genes has been used in wheat breeding, and frequently observed to be short-lived [4,9]. Almost all Lr genes from wheat origin present in the germplasm developed in Argentina in recent decades are, or have been, ineffective to one or more races of the pathogen, and the same occurred with the use of individual genes from species related to wheat, e.g., *Lr9*, *Lr19*, *Lr24* and *Lr26* [11]. Despite this situation, some wheat varieties remained resistant for a long time. This kind of resistance was defined as “durable” by Johnson [12]. Host genetic analysis carried out on durable varieties revealed that some combinations of major seedling resistance genes, known as All Stage Resistance (ASR) and Adult Plant Resistance (APR) genes, conferred resistance over long periods of time, in different environments and against diverse pathotypes of the fungus [4,13,14,15,16,17,18]. A common feature of durable resistance varieties is the presence of APR genes, suggesting that their presence plays an important role in this kind of resistance. 

Some traditional Argentinean wheat varieties, such as Sinvalocho MA and Buck Manantial, showed durable resistance. Both cultivars have been used as leaf rust resistance source in many breeding programs in Argentina, North America and Eastern Europe [19,20,21,22]. To identify the genetic components of durable leaf rust resistance in wheat, crosses can be made between parents that show genetic variability/contrasting parents to obtain F1 hybrids, and then develop different types of populations. Recombinant Inbred Lines (RILs) populations have the advantage of homozygosity, and the possibility to test them in field evaluations under natural population infections in different environments and in greenhouse inoculations using leaf rust races at the seedling and the adult plant stage [17,23,24,25]. Following this approach, two crosses were carried out to study Sinvalocho MA (SV): SV × Gama6 (G6) and SV × PurpleStraw (P). Three genes were identified and associated with resistance in field testing to the pathogen’s natural populations in SV × G6, allowing the evaluation of different gene combinations and their individual contribution to the observed resistance: APR genes *LrSV1* and *LrSV2* and the ASR gene *LrG6* [17]. In SV × P, the APR gene *LrcSV2*, complementary to *LrSV2*, was detected [26]. In Buck Manantial (BM), genes *Lr3*, *Lr16* and *Lr17* were previously identified by Dyck et al. [21], who also suggested the presence of the adult plant resistance gene *Lr13*. Saione et al. [27] confirmed the presence of genes *Lr3*, *Lr16* and *Lr17*, and an unidentified additional seedling resistance gene.

Leaf rust resistance genes can be introgressed in wheat by using pathogen-specific races combined with marker-assisted selection (MAS), facilitating the analysis in segregating populations. The availability of markers for each resistance gene allows identifying them simultaneously in each individual, independently of gene epistatic effects. The genetic recovery of recurrent parent background by MAS can also reduce the number of backcrossing cycles in the resistance introgression process. This methodology is based on selecting the largest number of markers similar to recurrent parent alleles. In the Australian wheat breeding program, MAS recurrent parent background reduced to a half the number of backcross cycles [28,29,30,31,32]. In recent years, the availability of markers to assist selection of characters of agronomic importance in wheat has grown exponentially, including not only SSRs (Simple Sequence Repeats), DARTs (Diversity Array Technologies) and SNPs (Single Nucleotide Polymorphisms), but also complete or partial genome sequences of different wheat cultivars, wheat ancestors and related cereals such as barley, rice, *Brachypodium* sp., sorghum, etc. (https://urgi.versailles.inra.fr/ (accessed on 30 August 2021)).

The paper published by Vrana et al. [33] reporting the isolation of chromosome 3B of wheat by flow cytometry sorting was the starting point for the construction of large-insert chromosome-specific DNA libraries to simplify the analysis of the complex wheat genome. Chromosome 3B is the largest chromosome of wheat and its DNA sequence was the first published in a series that later included all wheat chromosomes [34,35]. This information encouraged our group to initiate fine mapping of two of the APR genes identified in Sinvalocho MA located on chromosomes 3B and 4B [17,26,36].

The objectives of the present study were: the identification of genetic components of leaf rust resistance in Buck Manantial; the fine mapping of APR genes *LrSV2* and *LrcSV2*; the introgression and pyramiding of resistance genes for leaf rust from Buck Manantial and Sinvalocho MA in seven susceptible commercial wheat varieties and marker-assisted selection of recurrent parent background to shorten the selection process.

## 2. Materials and Methods

### 2.1. Plant Material

#### 2.1.1. Genetic Components of Leaf Rust Resistance in BM

A population of 118 F8 recombinant inbred lines (RILs) was developed by a single-seed descendent from a cross between Buck Manantial (http://wheatpedigree.net/sort/show/8869 (accessed on 16 August 2021)) and the susceptible Purplestraw (http://wheatpedigree.net/sort/show/14252 (accessed on 16 August 2021)), to characterize genetic components for leaf rust resistance in BM and linked molecular markers. Since its release in 1965, this variety has remained resistant in large-scale cultivation, reaching 15% of the cultivated area in 1972 [11]. At present, it still behaves as resistant, but it is only sown marginally at the southern border of the province of Buenos Aires (Argentina). Seedling resistance genes *Lr3*, *Lr16* and *Lr17* were identified in BM [21,27] and the presence of the APR gene *Lr13* was suggested by Dyck [21]. P was used for more than 70 years in Argentina, both as a susceptible control in field tests and as susceptible parent in different crosses to study leaf rust resistance. The 118 F8 RIL population from BM × P was used for disease phenotyping at greenhouse, both at the seedling and flag leaf stage using different races of *P. triticina* to identify leaf rust resistance genes (Table 1). Ninety-four of them were genotyped and used for linkage map development.

#### 2.1.2. Fine Mapping of APR Genes *LrSV2* and *LrcSV2*

For fine mapping of genes *LrSV2* and *LrcSV2*, an F2 population of 3403 individuals from the cross SV × G6 and a 1292 F2 population from the cross SV × P were used, respectively.

#### 2.1.3. Introgression and Pyramiding of Resistance Genes for Leaf Rust from BM and SV in Seven Susceptible Commercial Wheat Varieties

Buck Manantial (BM) was used as a chromosome 2BS donor, carrying the ASR gene *Lr16*, APR gene *LrBMP1* (identified as the main responsible for leaf rust resistance in field tests during this work) and APR gene *Lr13*, postulated by Dyck [21].

Line R46 was used as donor of the APR genes *LrSV1* and *LrSV2* + *LrcSV2* and the ASR gene *LrG6*. This line was selected from the RIL population previously used for mapping resistance genes, derived from the cross Sinvalocho MA (http://wheatpedigree.net/sort/show/57739 (accessed on 16 August 2021)) and the experimental line Gama6 (G6) [17]. G6 was obtained from SV by gamma-ray treatment and used in genetics studies for leaf and stem rust resistance [37]. The ASR gene *LrG6* was identified in this line, and observed to contribute to resistance in field tests [17].

**Figure 1:**
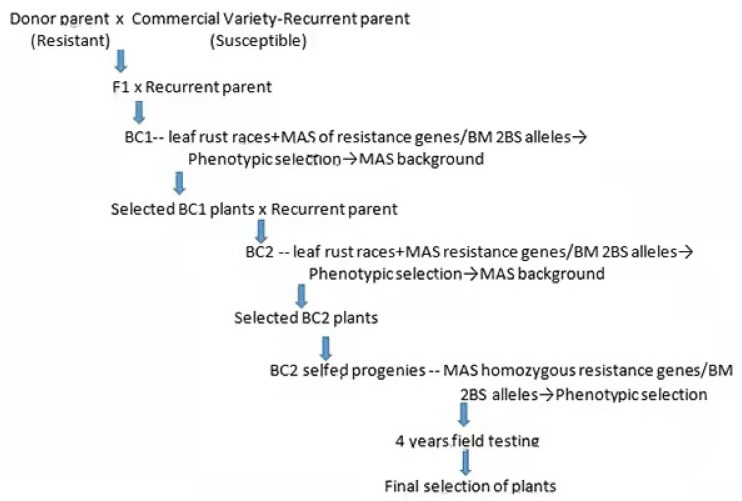
Scheme of introgressions.

The seven commercial varieties used as recipients in the introgressions were: ACA801, Baguette9, BioINTA1001 and Buck Biguá, that were crossed to BM, and Klein Don Enrique (KDE), Onix and Relmo Sirirí (R.Siriri), crossed to R46. These varieties were selected because of their susceptibility to leaf rust, high yielding, intermediate to short cycle and good baking quality. The crosses and selfings were performed in a greenhouse, where individual plants were grown in 3 **L** pots. After every cycle of marker-assisted selection, a visual phenotypic selection of plants was made by resemblance to the original recurrent parent, considering height, plant architecture, stem thickness and spike size (Figure 1).

### 2.2. Field Testing

#### 2.2.1. Field Testing of F8 RIL Population from BM × P

The RIL population from the cross BM × P was exposed to natural pathogen populations (without infector rows) in eight field trials in three different locations in Argentina where wheat leaf rust is endemic and occurs yearly: Reconquista (Rq, 29° S–60° W) and Maciel (Ma, 32° S–60° W), both in the province of Santa Fe and Castelar (Ca, 34° S–58° W) in the province of Buenos Aires. These three locations are representative of different wheat agro ecosystems in the central plain of Argentina, the Pampas Region. This flat area is usually considered as an epidemiological unit for leaf rust disease, together with Uruguay and South Brazil [38]. Tests were carried out during 4 years in the experimental field of the Institute of Genetics at Castelar, 3 years in Maciel and 1 year in Reconquista. Ten to twelve seeds for each RIL were sown and grown in 1m rows without replicas. However, all RILs carrying *LrBMP1* gene were considered as replicas in each year and place Infection data score was based on the scale of Mains and Jackson [39], also described in McIntosh et al. [9], and infection severity was estimated counting the number of pustules per square centimeter on five independent flag leaves in each row. F-tests to compare variances and *t*-tests to compare means of numbers of pustules were performed between all RILs carrying *LrBMP1* gene versus all RILs without it. Comparisons were made for each year and place (Ca04, Ca05, Ca06, Ca07, Ma06, Ma07, Ma09, Rq06), places (Ca04 + Ca05 + Ca06 + Ca07, Ma06 + Ma07 + Ma09) and all years and places (Ca04 + Ca05 + Ca06 + Ca07 + Ma06 + Ma07 + Ma09 + Rq06).

#### 2.2.2. Field Testing of Resistant Selected Lines

To validate the effects of resistance genes, and background selection of the recurrent parents, field testing was carried out under natural infection conditions for four years in the experimental field at Castelar (34° S–58° W) in the province of Buenos Aires, Argentina. Selected resistant lines from each cross, including the original susceptible varieties as controls, were grown in 1 m rows (10–15 seeds for each line), and scored for leaf rust resistance at flag leaf stage according to the scale of Mains and Jackson [39], also described in McIntosh et al. [9]. In addition, plants most similar to the original varieties were selected considering characters such as height, plant architecture, stem thickness and spike size.

### 2.3. Rust Inoculations

Plants were grown at the greenhouse and infected by artificial inoculations, both at seedling (first to second leaf) and adult stage (flag leaf at ear emergence), by using different races of *Puccinia triticina (*Table 1). Infections were carried out spraying a suspension of 20 mg of urediospores in 50 mL of water with one drop of Tween 20. Incubation was performed in moist chambers (100% humidity at 15–25 °C) for 16 h. Afterwards, plants were kept in the greenhouse at temperatures that ranged between 15 and 25 °C. Reactions were scored after 12–14 days for seedlings and 14–21 days for adult plants, according to Mains and Jackson’s scale [39], also described by McIntosh et al. [9].

### 2.4. DNA Markers

Genomic DNA was isolated according to Sacco et al. [41]. AFLP and PCR reactions and electrophoresis were performed according to Ingala et al. [17]. AFLP products were visualized by silver staining as described previously [42] and the rest of the markers were stained by the silver/NaOH method [43]. PCR primers and cycling conditions were as described for gwm [44], barc [45], wmc [46], gpw [47], cfb and cfp [48], sts [49], stm [50], gdm [51], psp [52] and swm [53]. 

#### 2.4.1. Mapping Resistance Genes in BM

A genetic linkage map, including leaf rust resistance genes identified in BM, was developed with 94 F8 RILs from BM × P, using 530 molecular markers (173 SSR and 357 AFLPs). This map was constructed using JoinMap v3.0 [54]. 

#### 2.4.2. Fine Mapping of Resistance Genes LrSV2 and LrcSV2

Insertion site-based polymorphism (ISBP) markers were designed with IsbpFinder software, as described by Paux et al. [55]. PCR products were purified in Mo-Bio columns and sequenced in an automatic capillary sequencer. The design of SSR markers was carried out using the program WEBSAT [56] and named FSs (listed in Supplementary Tables S1 and S2).

#### 2.4.3. MAS for Leaf Rust Resistance Genes Introgression and Background MAS

Selection of the ASR gene *LrG6* was performed using races of the pathogen (Table 1). The selection of BM chromosome 2BS was performed by selecting BM alleles in selected SSRs from this chromosome arm (Table 2). For Background MAS, recurrent parent alleles in at least 1 or 2 independent markers for each chromosome arm were selected. Different sets of markers were used in each backcross cycle considering the replacement of those markers that in BC1 were fixed in a homozygous condition. In BC1 and BC2, 21–36 and 28–34 SSRs were assayed, respectively. For KDE, where an additional cycle of selfed BC1 was introduced, 42, 40 and 55 SSRs were assayed in BC1, selfed BC1 and BC2, respectively (Supplementary Tables S3 and S4).

For the other introgressed genes, either SSRs or AFLPs were used (Table 3).

#### 2.4.4. Selection of Resistant Lines

After BC2 plants were selected, selfed progenies were obtained and homozygous resistant genes were selected using races or linked markers.

### 2.5. Data Analysis

Chi-squared tests were used for goodness of fit and independence of segregation for leaf rust resistance genes, and an *F*-Test for differences in number of pustules/cm^2^ at the flag leaf stage for the F8 RIL population from BM × P.

The percentages of the recurrent genome (RG%) were calculated according to the methodology used by Benchimol et al. [57]. The formula RG% = [B + (0.5H)/(B + H + A)] × 100 was used, where B corresponds to the number of alleles of the recurrent parent type, A to the number of alleles of the donor type parent and H to number of heterozygotes.

## 3. Results

### 3.1. Genetic Mapping of Leaf Rust Resistance Genes in Buck Manantial

Three selected races from the collection stored at the Instituto de Genética “Ewald A. Favret” (INTA) *P. triticina* collection, were used to inoculate the 118 F8 RILs population from the cross BM × P and Thatcher near-isogenic lines at seedling stage to characterize ASR genes in BM (Table 1). At the seedling stage, Ma04BuckGuapo (avirulent on Tc*Lr16*) detected one gene (Pχ^2^1:1 = 0.92), Rq05Cronox (avirulent on Tc*Lr16* and Tc*Lr17*) detected two genes (Pχ^2^3:1 = 1) and the race 66 (avirulent on Tc*Lr3*, Tc*Lr16* and Tc*Lr17*) detected three genes (Pχ^2^7:1 = 0.66). Race Ca02Lr17 was virulent at the seedling stage but detected an APR gene at the adult stage, temporally named *LrBMP1* (Pχ^2^1:1 = 0.47). Four recombinants were found between *Lr16* and *LrBMP1*, indicating a genetic distance of 1.7 cM (Chi^2^ independence test *p* < 0.0001).

**Figure 2:**
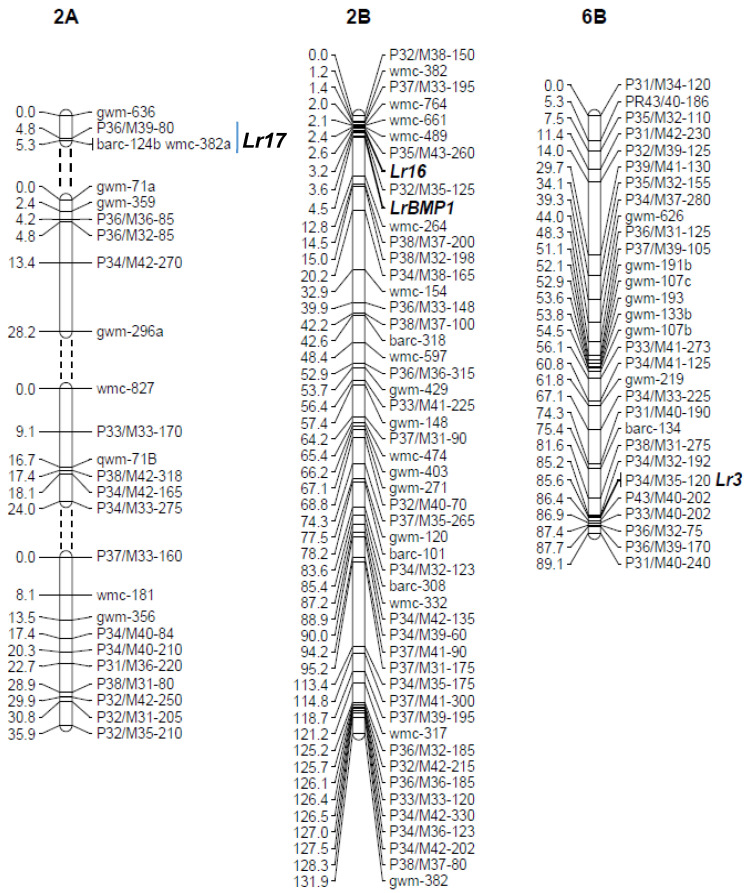
Linkage group of chromosomes 2A, 2B and 6B. On the left, cumulative genetic distances in cM. On the right, leaf rust resistance genes are shown in bold together with molecular markers. AFLPs were designated according to the primers used (*Pst*I primer/*Mse*I primer) and the size of the band in base pairs estimated from the mobility of molecular size markers run on the same gel.

A genetic linkage map of 173 SSR and 357 AFLPs (530 markers in total) was developed using 94 RILs from this population. The APR gene *LrBMP1* gene was mapped on chromosome 2B, and this chromosome was saturated with additional molecular markers, 30 AFLPs and 19 SSRs in total (Figure 2 and Supplementary Table S5). As previously reported, *Lr16* also mapped on distal 2BS [58]. For genetic mapping of *Lr17* and *Lr3* genes, RILs susceptible to Ma04BuckGuapo and Rq05Cronox were used, respectively, to obtain the phenotypic data. *Lr17* mapped on 2AS distal end [9] and *Lr3* mapped on the distal end of chromosome 6BL [42]. The presence of the *LrBMP1/Lr16* cluster showed a strong association to the leaf rust resistance observed under natural infection conditions in eight field trials in three different locations over four years. The average number of pustules per square centimeter was tested for all RILs carrying *LrBMP1/Lr16* cluster gene versus RILs without it in the eight trials. All means comparisons were highly significant (Table 4, Supplementary Table S6). According to Ingala et al. [17], less than 40–50 pustules/cm^2^ are indicative of moderate resistance. These authors also found that the infection type observed for that number of pustules or less were 0;122+, according to the Mains and Jackson scale for leaf rust [39], also described by McIntosh et al. [9].

### 3.2. Fine Mapping of LrSV2 and LrcSV2

Fine mapping allows the precise positioning of genes within chromosomes, facilitating their use for assisted introgression by closely linked markers. In addition, the development of this type of maps is a prerequisite to address positional cloning. For this purpose, flanking markers for two complementary APR genes previously detected in Sinvalocho MA, *LrSV2* and *LrcSV2,* were used to screen for recombination events within each target interval among large segregating populations.

**Figure 3:**
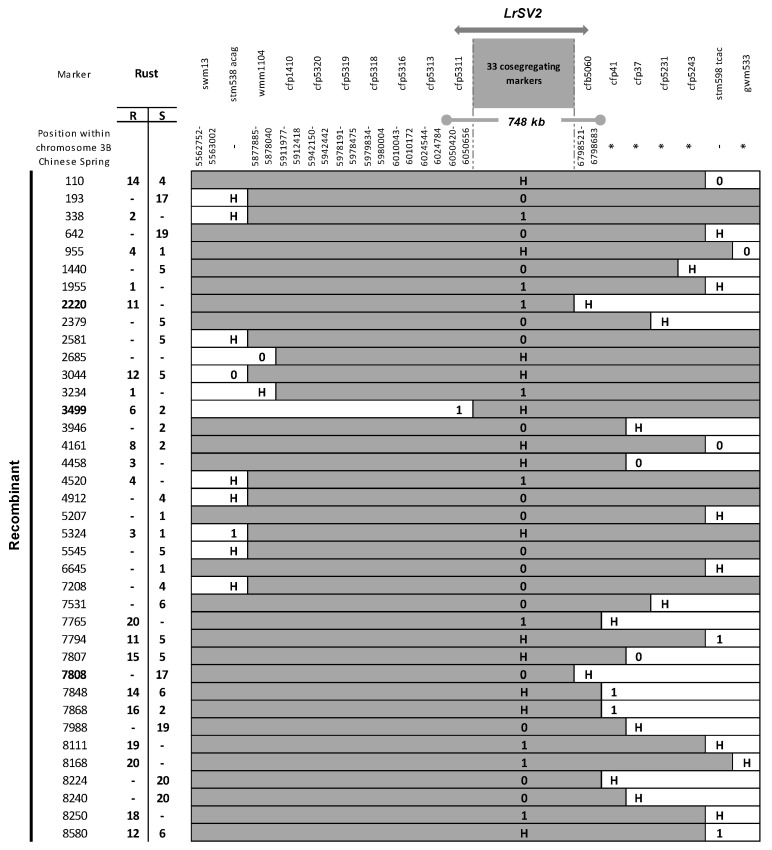
Graphical genotype table of the 38 critical recombinant F2s found between swm13 and gwm533 markers. Markers positions on the Chinese Spring chromosome 3B IWGSC RefSeq v2.1 is shown. * No blast on this 3B pseudomolecule, ordered according to conting344 [60]. Recombinants for the minimal interval containing the *LrcSV2* gene are depicted in bold. 0 susceptible Gama6 genotype, 1 resistant Sinvalocho genotype, H heterozygote genotype. Rust: F3 artificial infection (F4 for recombinant 642) with *P. triticina* race Ca02G1R at adult stage, R resistant phenotype, S susceptible phenotype. Cosegregating markers are described in Supplementary Table S1.

*LrSV2* flanking markers swm13 and gwm533 [36] were used to screen additional F2s from the cross SV × G6. In total, 38 recombinants were identified among 3403 F2s. Molecular markers reported to map on distal 3BS and new ones developed from the available CS chromosome 3B sequence contigs mapped to this interval [59] were evaluated for polymorphism. Out of 71 markers, 39 were polymorphic (Supplementary Table S1). These polymorphic markers, along with another 9 markers that cosegregated with the *LrSV2* gene [36], were used to determine the crossover position in each recombinant. After the phenotypic evaluation of the F3 progeny of these recombinants by artificial inoculation at flag leaf stage with the rust strain Ca02G1R, which detects the presence of the *LrSV2* gene in this cross, it was deduced that this resistance gene is located within an 0.04 cM genetic interval delimited by markers cfp5311 and cfb5060. These markers define a 748 kb interval on the physical map of CS (Figure 3).

**Figure 4:**
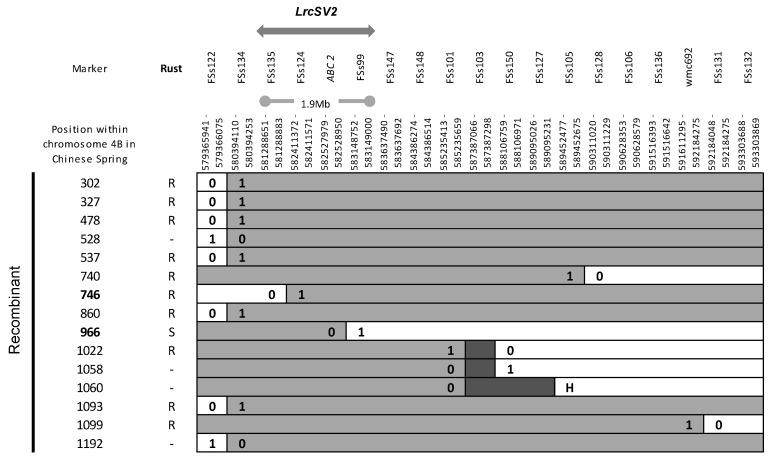
Graphical genotype table of recombinants found between FSs122 and FSs132 markers. Due to missing data in recombinants 1022, 1058 and 1060, possible crossover positions are denoted with hatched cells. Markers positions on the Chinese Spring chromosome 4B IWGSC RefSeq v2.1 is shown. Recombinants for the minimal interval containing the *LrcSV2* gene are depicted in bold. 0 susceptible Purplestraw genotype, 1 resistant Sinvalocho genotype, H heterozygote genotype. Rust: Artificial infection with *P. triticina* race Ca02G1R at adult plant stage, R resistant phenotype, S susceptible phenotype.

For *LrcSV2*, a stepwise approach was used. The first 584 F2s from the cross SV × P were genotyped with the flanking markers gpw4388 and FSs34 [26] and 9 recombinants were detected. At the same time, 44 additional markers within this interval were developed: 8 using the wheat 4B contigs spanning the target region identified as described in Diéguez et al. [26] and 36 using the sequence of the CS homologous region (https://wheat-urgi.versailles.inra.fr/Seq-Repository/Assemblies (accessed on 16 August 2021)). A total of 35 of them amplified a defined product of the expected size and 21 were polymorphic and associated with the gene by Bulk Segregant Analysis (Supplementary Table S2). The recombinants were genotyped with these markers, and their F3 was phenotypically assessed at flag leaf stage for resistance or susceptibility to the Ca02G1R rust strain, which detects *LrcSV2* in this cross. This analysis allowed the reduction in the *LrcSV2* interval between markers FSs132 and FSs122. These last two markers were used to screen 708 additional F2s (1292 in total). Recombinant analysis with internal markers and rust phenotyping defined a smaller interval between markers FSs135 and FSs99. These two markers delimit a region of approximately 1.9 Mb on the CS chromosome 4BL (Figure 4).

### 3.3. Introgression and Pyramiding of Leaf Rust Resistance Genes

The different stages of introgression were: crosses to donor resistant parents; backcrosses (BC) to recurrent susceptible parent; selection using specific races/MAS for introgressed genes and phenotypic selection; background MAS and final selfing to render homozygous genes from donor and recurrent parents (Figure 1)

Concerning KDE, an additional cycle of BC1 selfing was introduced because an unusual hail fall destroyed the crosses created for BC2 and only the recovery of some BC1 selfed progenies was possible.

Gene *Lr16* and linked *LrBMP1* were introgressed into the susceptible commercial varieties ACA801, Baguette9, BioINTA1001 and Buck Biguá. The presence of APR *Lr13* in BM was suggested by Dyck [21] However, we could not confirm this since avirulent races to this gene were not available in our collection. *Lr13* was mapped on 2BS on deletion bin 2BS1-0.53–0.75, proximal to the *LrBMP1*-*Lr16* cluster [61]. Therefore, markers throughout this chromosome arm chosen from a high-density microsatellite consensus map for bread wheat were used for its introgression (Table 2). In each BC cycle, plants were evaluated at seedling stage using the rust race Ma07Bg9 that identifies *Lr16* and/or the wmc764 linked marker and the selected plants were genotyped with 4 to 9 polymorphic codominant markers distributed along 2BS to select BM alleles.

Selected BC2 plants were selfed and their progenies were evaluated with markers on 2BS, to finally select those individuals with the greatest number of markers homozygous for the BM alleles (Table 5).

The ASR gene *LrG6* identified in G6 and the APR genes *LrSV1*, *LrSV2* and *LrcSV2* (complementary to *LrSV2*) identified in SV were simultaneously introgressed into the varieties Klein Don Enrique (KDE), Onix and Relmo Sirirí (R.Siriri). In each BC cycle, *LrG6* was selected using the corresponding rust race; *LrSV1* and *LrSV2* were selected by the associated molecular markers gwm261 or P31/M42 and gwm533 or P31/M37, respectively, and *LrcSV2* was tested in selfed BC2 by its associated marker gwm149 (Table 6 and Table 7). Noteworthy, the complementary effect of *LrcSV2* on *LrSV2* was only discovered after the introgressions were started [26]. However, the availability of a closely linked marker allowed the selection of lines harbouring this gene on selfed BC2s.

In the cross R46 × KDE, the four selected plants from the BC2 of selfed BC1 (Table 7) were selfed and only 55 seeds were obtained (a number not large enough to select homozygous plants for all the introgressed genes). For this reason, it was advanced to a new generation and progeny tests were performed on the 55 families obtained (12–13 plants per family) using the rust race Ca04KDE that is avirulent on *LrG6.* This allowed to identify 16 homozygous resistant families for the *LrG6* gene (χ^2^ 1:2:1 *p* = 0.5–0.3). Eighty-three plants were selected from these 16 families and were evaluated with the markers linked to *LrSV1* and *LrSV2*, selecting eight plants homozygous for the three introgressed resistance genes (χ^2^ 1:15 *p* = 0.20). These plants were evaluated with the marker gwm149, linked to *LrcSV2*, and all of them were homozygous.

The variety KDE carries *Lr26*, derived from the rye translocation 1B/1R [62]. Associated molecular markers have been reported for this resistance gene, as the dominant SCAR SCSS30.2 [63]. This SCAR was present in the eight selected plants; therefore, all of them carry at least one allele of *Lr26.* The progeny of these eight plants were tested during four years in the field at Castelar, and finally 5 lines were selected according to rust resistance, phenotypic characteristics by comparison with the original commercial variety and homogeneity (Table 7).

In addition, phenotypic characteristics of height, plant architecture, stem thickness, leaf color (only for Baguette9) and spike size were visually evaluated by comparing these attributes with the original commercial variety. This phenotypic characterization was prioritized with respect to %RG for selection; therefore, plants phenotypically different from the recurrent parent were discarded (Table 5, Table 6 and Table 7).

### 3.4. Field Testing of Introgressed Resistant Lines

**Figure 5:**
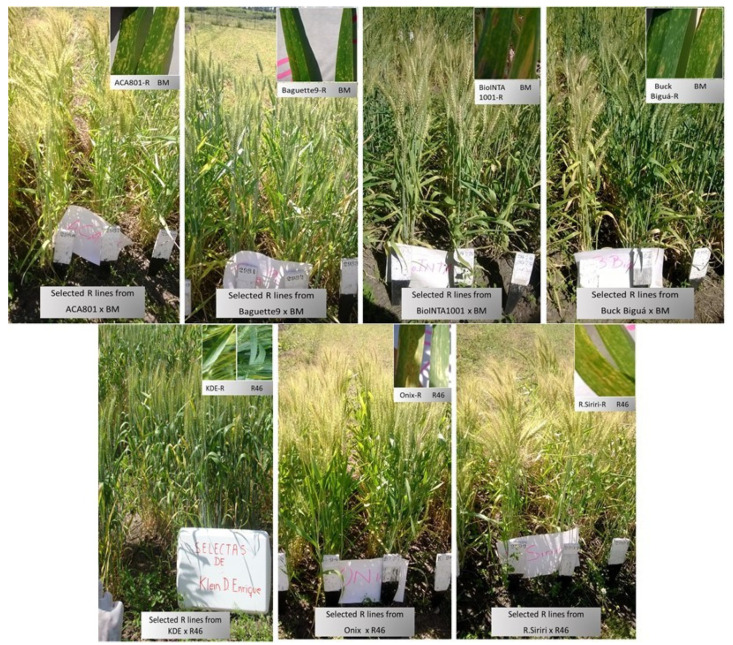
Selected resistant lines at field and detail of infected flag leaves from the introgressed line (left) compared with the resistance donor (right).

The progenies of BC2 selfed selected plants (or BC2F3 in the case of KDE) derived from each crosss were evaluated for leaf rust resistance at the experimental field of the Instituto de Genética “Ewald A. Favret” (INTA) for four years. A selection for leaf rust resistance and phenotypic traits was performed during the first two years, discarding those lines that showed phenotypic differences with the recurrent parent or heterogeneity. From the third year, and also during the fourth year, selected lines showed stability, obtaining the final number of lines indicated in Table 5, Table 6 and Table 7 (Figure 5). 

## 4. Discusion

### 4.1. Identification and Introgression of Genetic Components of Durable Leaf Rust Resistance

Traditional breeding is based on obtaining segregating populations and a further selection for the best combination of genes. However, to make this process efficient, it is important to know the genetic bases of the trait to be improved and unequivocally identify the genes involved. Durable resistance for wheat leaf rust has been reported in some varieties [14,17,64,65,66]. The study of the genetic components of the observed durable resistance present in a given variety can be carried out by disassembling the resistant genotype and identifying each component and interactions among them [17]. In this process, resistance genes can be identified even in lines such as Gama6 that was usually used as a susceptible parent but has shown resistance to some leaf rust isolates [37]. It is known that several APR genes are race-specific, such as *Lr12*, *Lr13* and *Lr22* [9], including *LrSV1*, *LrSV2* and *LrcSV2* used in this work that do not give a complete protection when used alone. There is also a range of unknown APR genes detected as QTLs, whose mapping is more complex but affordable using large RILs populations and saturated maps based on a large number of markers [66]. In contrast to the presence of single race-specific resistance genes alone, host genetic analysis carried out on durable varieties revealed that some particular combinations of ASR genes and APR genes (expressing minor or major effects) confer resistance over long periods of time, in different environments and against diverse pathotypes of the fungus [4,13,14,15,16,67]. Specific combinations involving APR genes *Lr13* and *Lr34*, and some seedling resistance genes showed enhanced resistance, both in greenhouse and field tests [15,68]. A common feature of varieties with durable resistance is the presence of APR genes, suggesting that they play an important role in this kind of resistance.

Another complexity posed by rust resistance is that the genes show epistatic effects and, in some cases, complementary effects that may be hidden without proper genetic analysis. Genetic studies are frequently performed by a single contrasting cross, but the analysis of different crosses might uncover genetic interactions between unlinked genes as observed for the complementary system *LrSV2 + LrcSV2* in Sinvalocho MA [36]. In breeding for rust resistance, the incorporation and selection of single genes is relatively simple [69,70] and can be carried out in early generations. However, as mentioned above, durable resistance sources present more complex genotypes, and the challenge is to introgress several genes without modifying other traits of interest in the final product. The strategy of pyramiding and selecting genes, both ASR and APR genes, and even minor genes [71], can be carried out by backcrosses using marker-assisted selection (MAS) of the introgressed gene and validated QTLs. Additionally, the genetic background of the recurrent parent can be enriched by marker-assisted selection (BC-MAS). In some instances, the outbreak of diseases in areas where most of the varieties revealed as susceptible, or where the disease did not exist before, poses the challenge of rapidly converting commercial susceptible varieties into resistant ones. For this purpose, MAS and BC-MAS are especially suited.

In the present work, two sources of resistance (BM and R46) were used to convert seven commercial susceptible varieties in resistant ones. BM was used as a donor of chromosome 2BS carrying genes *LrBMP1*, *Lr16* and putatively *Lr13*. R46, an F8 RIL derived from the cross SV × G6, was used as a donor of *LrSV1*, *LrSV2, LrcSV2* and *LrG6* by using two backcrosses and two selfed backcrosses progenies (BC2F2 for six varieties and BC2F3-F4 for KDE) in a period of five years, including 2 years of field testing and selection. Two additional years to test the stability of selected resistant lines may be considered. Two generations per year were carried out in a standard greenhouse using strains of the pathogen and robust markers such as SSRs, both to assist the selection of resistance genes (foreground) as the genetic background. Moullet et al. [72] reported the introgression of three resistance genes to leaf rust after seven backcrosses and one final selfed generation during 6 years. This author used markers linked to gene *Lr9*, *Lr24* and *Lr22a* to confirm their presence in selected resistant plants. The availability of detailed studies about the genes present in both sources of resistance, BM and R46, and markers associated with them, facilitated the selection process through the backcrosses. The methodology described here allowed selecting in the field, under natural infection conditions for 4 years, 19 lines resistant to leaf rust (infection type R-RMR), very similar phenotypically to the original varieties used as susceptible controls, with %RG (percentage of recurrent genome) that ranged from >80 to >93%. Randhawa et al. [73] have reported 97% recurrent parent genome recovery after just two backcrosses in wheat, but used a different approach based on computer simulation and wheat genome structure information.

Durable resistance varieties usually showed low-intermediate levels of infection, and resistant/moderately resistant infection type (small pustules with necrosis and/or small to medium sized pustules with green islands and surrounded by necrosis or chlorosis), and in some intense attacks moderately resistant/moderately susceptible. In the present work, converted varieties carrying *LrBMP1*, *Lr16* and putatively *Lr13* from BM (11 lines) and those carrying genes *LrSV1*, *LrSV2*, *LrG6* and *LrcSV2* from R46 (8 lines), showed low levels of infection and low number of pustules, ranging from 10–20 pustules per square centimeter and infection type 0; 11 + (according to the scale of Mains and Jackson, [39]), also described in McIntosh et al. [9]. These levels of resistance were similar to those observed in the studies carried out to identify the genetic component of leaf rust resistance in SV [17].

The methodology proposed here allowed to introduce resistance genes using a traditional methodology, such as backcrossing, and assisted selection by robust DNA markers such as microsatellites (SSRs). The combination of both techniques allowed shortening the introgression cycles significantly, which results in cost savings, whereas improving selection efficiency is a critical factor in any improvement program. As the final product of the project, lines derived from the seven varieties mentioned above were achieved, with substantial improvement, since different leaf rust resistance genes were incorporated with proven effectiveness in their field behavior. The hypothesis of pyramiding resistance genes, both ASR and APR, is based on the fact that individual gene effects are additive, resulting in a broad protection against new virulences. It may be speculated that APR genes (major and minor) give a basal level of resistance and the simultaneous presence of ASR genes may improve this resistance [64,71]. In the work published by Ingala et al. [17], the effects of individual and combined genes were clearly demonstrated on improving resistance to natural pathogen populations. In that work, field testing was carried out in hot spot places for leaf rust as Maciel and Castelar in Central Argentina, and the fact that the genes tested come from durable resistance varieties, might increase the probability of producing resistant cultivars over the years, although there is no guarantee that such resistance would be durable. Under these circumstances, it is essential monitoring pathogen populations and develop methods to predict the occurrence of new virulent pathotypes [74].

In theory, 87% of the genetic background of the recurrent parent is expected after 2 backcrosses. This trend was clearly observed in the values observed in this work, since %RG ranged between 80 and 94%. However, the advantage of using markers with respect to the traditional methodology, is that it allows the knowledge of the real composition of selected genotypes and not in terms of probability. In addition, in selected lines with resistance genes already in a homozygous condition, for each additional generation of self-fertilization, the recurrent parent background homozygosity can be increased by 25% for those genes that are still in heterozygous condition. Finally, it is worth mentioning that the phenotypic observation of all traits of economic importance determines the commercial suitability of the rust resistant line. This phenotypic evaluation usually performed by breeders, is less costly that any molecular evaluation and allows to select at the same time many agronomic characters. Additionally, further characterization of flour, baking quality and protein content could be carried out to further select desirable traits.

The APR locus *LrBMP1* that confers highly effective resistance to natural leaf rust infection detected in BM is likely to be different from the previous APR QTLs mapped on chromosome 2BS QLr.hbu-2BS.1 and QLr.hbu-2BS.2 [75]. These QTLs are flanked by SSRs that are approximately 50 cM apart from the genetic position of *LrBMP1*, located on the telomeric region close to *Lr16* as compared in the wheat consensus map of Somers et al. [46]. *LrBMP1* genetic position is also different from APR *Lr13* position whose presence in BM was suggested by Dyck et al. [21] and was recently mapped on deletion bin 2BS1-0.53–0.75, proximal to the *LrBMP1-Lr16* cluster [61].

Other durable resistant varieties studied in our lab are Buck Poncho (BP) and El Gaucho FA (EG). In the RIL population BP × P, ASR genes *Lr10* and *Lr11* and one APR gene temporarily named *LrBP3* were identified [76]. However, the leaf rust response in the field showed that none of the gene combinations reached infection severities as low as BP, indicating that the three genes only partially explained the observed level of resistance in BP [76]. A saturated map of this cross was recently developed, and a QTL analysis is underway (Cuyeu AR, personal communication). A RIL population was also developed from the cross EG × P and at least two ASR genes were detected in EG (Sacco F, personal communication).

The identification of genes responsible for durable resistance to wheat leaf rust in traditional varieties currently in disuse due to their undesirable agronomic characteristics such as low yield, plant height, cane thickness, spike architecture, etc., would enable their use. These genes could be lost, as these varieties are no longer used in breeding programs. Many durable varieties for leaf rust are derived from old land races and varieties from Brazil, Uruguay and Argentina [67]. Its characterization, fine genetic mapping and finally its cloning would allow both the development of markers based on its nucleotide sequence for its 100% efficient monitoring, as well as its incorporation by genetic engineering or editing [64]. It would also provide knowledge for a better understanding of the molecular processes involved in resistance, as well as the design of novel strategies for crop protection [77].

### 4.2. Fine Mapping of Leaf Rust Resistance Genes Identified in Durable Varieties

Fine mapping consists of the identification of an accurate small genetic interval using a population with high number of segregating individuals. It is a prerequisite for positional cloning that will allow its rational use and the study of the underlying molecular action mechanism. This strategy was successfully used for map-based cloning of leaf rust genes *Lr1*, *Lr10*, *Lr21* and *Lr34* in which intervals of 0.8, 0.13, 1.7 and 0.15 cM, respectively, were defined [78,79,80,81]. These authors used markers developed from wheat ancestors or related species [80,81,82,83]. In the present work, we were able to develop fine maps taking advantage of the publicly genomic resources available at each time point: BAC-end sequences [48,60], contigs of the physical map of chromosome 3B of the reference cultivar CS [59] and finally the complete genome sequence of this wheat model variety [35].

It should be noted that through gel electrophoresis, ISBP (Insertion Site Based) markers allow the detection of presence⁄absence polymorphisms. However, the high methylation level of transposable elements (TEs) leads to an increase in mutation frequency at deaminated sites [84,85] resulting in a single nucleotide polymorphisms (SNP) frequency of one every 99 bases [55]. Therefore, in cases where the ISBP marker amplifies in both parents, it is still possible to detect SNPs by sequencing or by HRM analysis [55]. By sequencing markers cfp41 and cfp1410 (previously reported as non-polymorphic in the SV × G6 cross by Diéguez et al. [36]) and cfp5304, cfp5311, cfp5313, cfp5318, cfp5319, cfp5355 and cfp5358 in parental lines SV and G6 we found SNP polymorphism with a frequency of approximately 1/137 bp. Given the abundance of ISBP markers in the wheat genome, they can improve marker development even in crosses which are not very polymorphic as SV × G6.

In addition, by adjusting PCR conditions, some markers previously reported as not amplified or non-polymorphic in Diéguez et al. [36], were made polymorphic such as cfb5000, cfb5009, cfb5014, cfb5015, cfb5021, cfb5026 and cfp37.

The *LrSV2* interval defined here, delimited by markers cfp5311 and cfb5060, overlaps with the defined *Sr2* adult plant stem rust resistance locus on 3BS [86]. In certain backgrounds, *Sr2* is also associated with race-specific leaf rust seedling resistance due to *Lr27*, which is linked to *Sr2* [86,87]. The *P. triticina* race Ca02G1R which was used to identify *LrSV2* at adult stage was also avirulent on seedlings carrying *Lr27* + *31* [17]. However, while *Lr27* + *31* was active at the seedling stage, Sinvalocho MA behaved susceptible at the seedling stage, and *LrSV2* was not detected until later developmental stages such as the flag leaf, supporting the hypothesis that they are not the same gene.

Another common feature between *LrSV2* and *Lr27* is the need of a complementary gene on 4BL [9,26,88]. However, considering that *Lr31* is allelic or the same as *Lr12*, as suggested by Singh et al. [89], the position of *Lr31* and *LrcSV2* on 4BL is not coincident. *LrcSV2* gene, which is complementary to *LrSV2*, was already mapped within a 23 Mb interval that is 2 cM distal to the *Lr31* flanking markers [26]. In the present work, *LrcSV2* was further mapped within this interval to a region that corresponds to 1.9 Mb on the CS chromosome 4BL sequence.

In the final steps of map-based cloning, it is desirable to use a genomic library of the cultivar or line that contains the gene of interest, as the sequence corresponding to the target gene might be absent in the CS genomic sequence. Construction and organization of BAC libraries is laborious and costly, especially from organisms with large and complex genomes as wheat. However, a pooled BAC library strategy [90] would allow the rapid and low-cost generation and isolation of BAC clones spanning the region surrounding the gene of interest. A Sinvalocho MA BAC library was developed and is available for screening [91]. Recently, some alternative methods (e.g., MutRenSeq, TACCA, and MutChromSeq) have been used to clone wheat disease resistance genes [92,93,94].

The use of resistance genes, particularly those from varieties that show durable resistance, significantly reduces the use of pesticides, which is both cost-effective and environmentally sustainable and constitutes a significant contribution to improve the competitiveness of this crop, within a framework of climate change that could favor fungal diseases [95].

## 5. Conclusions

Resistance genes identified in durable resistant varieties can improve harvest safety and sustainability of modern varieties. Their introgression can be accelerated by use of associated molecular markers to assist both foreground genes selection and recipient genomic background selection. In the present work, in a period of five years, including 2 years of field testing and selection, two sources of resistance were used to convert seven commercial susceptible varieties in resistant ones. Two additional years to test the stability of selected resistant lines may be considered. Two generations per year were carried out in a standard greenhouse using strains of the pathogen and robust markers such as SSRs.

## Figures and Tables

**Table 1:** Leaf rust traces used in this work. * According to North American System [40].

Cross	Rust Race	Genes Detected	Resistance Type	Nomenclature *
R46 × KDE	Ca04KDE	*LrG6*	ASR	MCT
R46 × R.Siriri	Ma07Bg9	*LrG6*	ASR	MFT
R46 × Onix	Ma07Bg9	*LrG6*	ASR	MFT
BM × Baguette9	Ma07Bg9	*Lr16*	ASR	MFT
BM × ACA801	Ma07Bg9	*Lr16*	ASR	MFT
BM × Buck Biguá	Ma07Bg9	*Lr16*	ASR	MFT
BM × BioINTA1001	Ma07Bg9	*Lr16*	ASR	MFT
BM × P	Ma04BuckGuapo	*Lr16*	ASR	MBT
BM × P	Rq05Cronox	*Lr16 + Lr17*	ASR	MDR
BM × P	66	*Lr3 + Lr16 + Lr17*	ASR	JBB
BM × P	Ca02Lr17	*LrBMP1*	APR	MHP
SV × G6	Ca02G1R	*LrSV2*	APR	MCG
SV × P	Ca02G1R	*LrcSV2*	APR	MCG

**Table 2:** SSRs used for Buck Manantial chromosome 2BS selection. ^1^ used only in BC1 ^2^ linked to *Lr16* (Figure 2).

BM × ACA801	BM × Baguette9	BM × BioINTA1001	BM × Buck Biguá
barc124 ^1^	barc18	gwm148	barc124
gwm148	gwm374	gwm374	gwm257
gwm257 ^1^	gwm403	wmc597	gwm374
gwm374	wmc597	wmc764 ^2^	gwm630
gwm403	wmc764 ^2^		wmc764 ^2^
gwm630			
gwm636 ^1^			
wmc597 wmc764 ^2^			

**Table 3:** Molecular markers used to assist the selection of the indicated gene.

Gene	Resistance Type	Associated Marker
*LrSV1*	APR	gwm261/P31/M42
*LrSV2*	APR	gwm533/P31/M37
*LrcSV2*	APR	gwm149
*Lr16*	ASR	wmc764
*Lr26*	ASR	SCAR SCSS30.2

**Table 4:** Mean number of pustules per square centimeter in field trials of RILs with *LrBMP1* presence vs. absence. Ca: Castelar, Ma: Maciel and Rq: Reconquista.

Place/Year	Mean Number of Pustules/cm^2^ *LrBMP1* Presence vs. Absence	*t*-Tests *p* Value
Ca04	34.8 vs. 69.7	*p* > 0.001
Ca05	41.5 vs. 70.6	*p* > 0.001
Ca06	29.8 vs. 61.4	*p* > 0.001
Ca07	33.7 vs. 67.3	*p* > 0.001
Ma06	38.1 vs. 57.4	*p* > 0.001
Ma07	32.2 vs. 62.7	*p* > 0.001
Ma09	30.6 vs. 60.2	*p* > 0.001
Rq06	45.4 vs. 66	*p* > 0.001
Ca04 + Ca05 + Ca06 + Ca07	35 vs. 67	*p* > 0.001
Ma06 + Ma07 + Ma09	33.6 vs. 60.1	*p* > 0.001
Ca04 + Ca05 + Ca06 + Ca07+ Ma06 + Ma07 + Ma09 + Rq06	36.1 vs. 64.1	*p* > 0.001

**Table 5:** Introgression into ACA801, Baguette9, BioINTA1001 and Buck Biguá. Number of plants selected in each cycle. ^1^ Selected by seedling resistance to *P. triticina* race Ma07Bg9, ^2^ Selected by its associated marker wmc764, ^3^ Selected by genotyping codominant markers distributed on 2BS, ^4^ Percentage of recurrent parent genome, calculated as described in Materials and Methods, ^5^ Selected by rust resistance, phenotypic characteristics of height, plant architecture, stem thickness and spike size by comparison with the original commercial variety and homogeneity, ^6^ Minimum %RG according to the BC2 %RG, NT: Not tested.

Cross	BC1				BC2				Selfed BC2			Progeny of Selfed BC2	
	Total	*Lr16* ^1^	*Lr16*^2^ + BM-2BS ^3^	Selected (%RG ^4^)	Total	*Lr16* ^1^	*Lr16*^2^ + BM-2BS ^3^	Selected (%RG ^4^)	Total	*Lr16* ^1^	*Lr16*^2^ + BM-2BS ^3^	Final Field Selection ^5^	%RG ^6^
BM × ACA801	210	94	22	2 (71–77)	303	139	22	7 (80–86)	1115	299	19	3	>80
BM × Baguette9	138	75	22	5 (78–86)	337	177	19	8 (85–94)	1997	529	21	1	>85
BM × BioINTA1001	308	NT	22	6 (78–85)	474	NT	22	10 (83–90)	1734	428	11	4	>83
BM × Buck Biguá	283	139	19	5 (72–80)	409	207	22	7 (81–88)	2214	509	11	3	>81

**Table 6:** Introgression into Onix and R.Siriri. Number of plants selected in each cycle. ^1^ Selected by seedling resistance to *P. triticina* race Ma07Bg9; ^2^ selected by their associated markers gwm261 and gwm533, respectively; ^3^ percentage of recurrent parent genome, calculated as described in Materials and Methods; ^4^ selected by its associated marker gwm149; ^5^ selected by rust resistance, phenotypic characteristics of height, plant architecture, stem thickness and spike size by comparison with the original commercial variety and homogeneity; ^6^ minimum %RG according to the BC2 %RG.

Cross	BC1				BC2				Selfed BC2		Progeny of Selfed BC2	
	Total	*LrG6* ^1^	*LrSV1* + *LrSV2* ^2^	Selected (%RG ^3^)	Total	*LrG6* ^1^	*LrSV1* + *LrSV2* ^2^	Selected (%RG ^3^)	Total	*LrSV1*, *LrSV2*^2^, *LrG6*^1^ and *LrcSV2* ^4^	Final Field Selection ^5^	%RG ^6^
R46 × Onix	198	94	19	4 (79–87)	225	124	22	9 (85–96)	1458	19	2	>85
R46 × R.Siriri	215	106	21	2 (77–84)	143	75	16	8 (84–97)	997	8	2	>84

**Table 7:** Introgression into KDE. Number of plants selected in each cycle. ^1^ Selected by seedling resistance to *P. triticina* race Ca04KDE; ^2^ selected by their associated AFLP markers P31/M42 and P31/M37, respectively; ^3^ percentage of recurrent parent genome, calculated as described in Materials and Methods; ^4^ selected by their associated markers gwm261 and gwm533, respectively; ^5^ selected by its associated marker gwm149; ^6^ selected by its associated marker SCAR SCSS30.2; ^7^ selected by rust resistance, phenotypic characteristics of height, plant architecture, stem thickness and spike size by comparison with the original commercial variety and homogeneity; ^8^ minimum %RG according to the BC2 %RG.

Cross	BC1				Selfed BC1				BC2 of Selfed BC1				BC2F3		BC2F4	
	Total	*LrG6* ^1^	*LrSV1* + *LrSV2* ^2^	Selected (%RG ^3^)	Total	*LrG6* ^1^	*LrSV1* + *LrSV2* ^2^	Selected (%RG ^3^)	Total	*LrG6* ^1^	*LrSV1* + *LrSV2* ^4^	Selected (%RG ^3^)	Total	*LrSV1* + *LrSV2* ^4^, *LrcSV2* ^5^, *Lr26* ^6^	Final Field Selection ^7^	%RG ^8^
R46 × KDE	115	59	15	4 (37–52)	156	110	28	2 (65–71)	66	66	21	4 (93–96)	83	8	5	>93

## Data Availability

Not applicable.

## Supplementary Materials

The following are available online at https://www.mdpi.com/article/10.3390/biology10111168/s1, Table S1: Primer sequences of the markers within the swm13-gwm533 *LrSV2* interval. The length (in bp) of the amplified product in Sinvalocho (SV) and Gama 6 (G6) is indicated. Polymorphic markers are depicted in bold. * SNP: Single nucleotide polymorphisms. • cosegregating markers. Reference [96] are cited in the Supplementary Materials. Table S2: Primer sequences of the designed microsatellites within the gpw4388-FSs34 *LrcSV2* in-terval. The length (in bp) of the amplified product in Sinvalocho (SV) and Purplestraw (P) is indicated. Polymorphic markers are depicted in bold. Table S3: SSRs markers assayed for recurrent parent background selection in 2BS-BM introgressions. Table S4: SSRs markers assayed for recurrent parent background selection in R46 introgressions. Table S5: Genotypic matrix of 94 RILs from the cross Buck Manantial × Purplestraw. “A” and “B” alleles represent same allele as Buck Manatial and Purplestraw, respectively. AFLPs were designated according to the primers used (PstI primer/MseI primer) and the size of the band in base pairs estimated from the mobility of molecular size markers run on the same gel. Primers for gwm, wmc and barc SSRs were as described by Roder et al. [44], Somers et al. [46] and Song et al. [45], respectively. If more than one polymorphic band were observed for a given microsatellite, they were named with Indexes “a”, “b” and “c”. In sheets 2A, 2B and 6B, markers for these linkage groups, ordered according to the genetic map and their genetic positions. Table S6: Statistical analysis of number of pustules per square centimeter in eight field trials of the BM × P RILs population.
