# Supplementary material for: Characterization and Use in Wheat Breeding of Leaf Rust Resistance Genes from Durable Varieties
Source: Biology (Basel). 2021 Nov 12;10(11):1168. doi: 10.3390/biology10111168 (PMC8615195; doi:10.3390/biology10111168)
Supplement: Supplementary file 1 [file biology-10-01168-s001.zip › Table S1 - Markers LrSV2-interval.pdf]

Table S1: Primer sequences of the markers within the swm13-gwm533 *LrSV2* interval. The length (in bp) of the amplified product in Sinvalocho (SV) and Gama 6 (G6) is indicated. Polymorphic markers are depicted in bold. * SNP: Single nucleotide polymorphisms. • cosegregating markers (in addition to cfb5006/5007/5008/5010/5025/5061, gpw7080 and SCAR40/42 from [36]

	Forward primer	*Reverse primer*	*SV*	*G6*	*Marker type*	*Reference*
**cfb5000 •**	ATCCCCATCCCTCCTCCC	AGTCATTCATCCCGTTGCC	-	251	SSR	**Sourdille P., personal**
**cfb5009 •**	TCCCTCAACCCGTCGCTC	TTCTTCCTCCCGCTTCTGCTG	139	141	SSR	**Sourdille P., personal**
**cfb5011 •**	TGCAGCTACTCTATTACTCCTC	AGCACAAGCAAAGCAGAAAG	-	224	SSR	**Sourdille P., personal**
**cfb5013 •**	TTTTGAAGGCTTGGAGCAC	ATATTACACACACCGACACAC	-	259	SSR	**Sourdille P., personal**
**cfb5014 •**	CGTCCACTCCATTTACAGCAC	TGGAGCAGGGAGAGGAGG	-	234	SSR	**Sourdille P., personal**
**cfb5015 •**	TGGCCCATGCTGGCCTAC	GCGAAGCACAATGCGAATGAC	259	268	SSR	**Sourdille P., personal**
**cfb5018 •**	CGACGGAGGGGAATTTAGTAG	GCACAAAGAGAAGAGGGCAG	-	307	SSR	**Sourdille P., personal**
**cfb5019 •**	ATGCTCGTCCTACTCCCC	GTTACACACCCACACAACC	-	310	SSR	**Sourdille P., personal**
**cfb5021 •**	GCGCTAGATTTTTCAGTGACC	CACAAATATCATAGTGCCCTCC	347	351	SSR	**Sourdille P., personal**
**cfb5023 •**	TCAATCCTCCCTCCATCAAC	GGCAGAAGAAACGGAAGC	-	225	SSR	**Sourdille P., personal**
**cfb5026 •**	CTGGAATGTGTGCTGAAAATC	GCCTTTGAAATTAAGTCAACCC	306	-	SSR	**Sourdille P., personal**
**cfp37**	ATGATCTTGCGTGTGCGTAG	TGACAAATTCTCGCAGCAAC	170	-	ISBP	**[60]**
**cfp41**	CATGCTCGTCATCGAGTAGG	GGCACTGCTGATAGCCTC	290*	290*	ISBP-derived SNP	**[60]**
**cfp1410**	GGAAGGTGAGGCATGATCCAAAG	CCCACTGCCGGGAGTGAG	442*	442*	ISBP-derived SNP	**[60]**
cfp5200	TTCACACGGCTTGTCTTGTC	AAAAGCGTCTCGATCACTACTC	189	189	ISBP	this work
cfp5205	AGTGCTGTGCTGTGCTGTTT	GTACTCGTCCGCTTCACTCC	300	300	ISBP	this work
cfp5207	TTGGAATTTCCGGTTCACAT	GAGGCTCACTAGGGACATGG	219	219	ISBP	this work
cfp5208	CCGCTTAGCGATCTACAAGG	GGTTTTGATATCTGTCCCCG	222	222	ISBP	this work
cfp5209	AGGCAATCGATGGTGGTTAC	AAAAATCGCCTATTCACCCC	266	266	ISBP	this work
**cfp5222 •**	TTTGCTTCATCACCCTTTCC	GAACCGTTTGCGATGAAAAT	-	271	ISBP	**this work**
cfp5223	GCAAACAGAAAAAGGCAAGC	ACAATTCTAGCATGGATAATAAACAA	266	266	ISBP	this work
cfp5224	GCGATGGTCTTTTCCTACCA	AACCGGCACTATTGTGCTTC	217	217	ISBP	this work
cfp5226	TGTTCGACTACCTCATCCCC	TTGCCTGGATCCATCAAAAT	219	219	ISBP	this work
cfp5227	ACCCTCGTGTCTTTGTGTCC	CGGTAAACGGGAGTTGCATA	210	210	ISBP	this work
cfp5229	CGGGATCACATCATTAGTTGAA	TCGGTCCATAGAGTCCCAAC	256	256	ISBP	this work
**cfp5231**	TAGTGGCAGTAACCGTGGTG	TTAACTTTCCGGTTTGCCTG	-	244	ISBP	**this work**
cfp5236	AATAAAACACCGAGGGGAGG	ATGCGAAAAACTCTGATGGG	241	241	ISBP	this work
cfp5237	TCATATCACGGGGTTTGGAT	ATGTAACCGGCACTACAGGG	127	127	ISBP	this work
cfp5241	CAGCCCCTACATTGGTGAAC	CATAATTCACTCGACCGCCT	237	237	ISBP	this work
**cfp5243**	CGACGAGCACTTTACCATCA	ACTAACCGAGGTGATGTGGC	-	203	ISBP	**this work**
cfp5254	CATCGTCCAGATGCAGAGG	CCACCCAACAAATGTGTCAA	137	137	ISBP	this work
cfp5263	ACAATAAGGGCACGACAAGG	ATATGGCAAGCAATCCAAGG	178	178	ISBP	this work
**cfp5300 •**	GGCTTGAAGCTTTGAACCAG	TCCTCCATTTTCAAATAAGTGTCTC	178	-	ISBP	**this work**
**cfp5301 •**	TCCCTCTGTCTCAAAATAAGTGTC	CCAATATCAGGAGTGCGGTT	190	-	ISBP	**this work**

cfp5302	CATCCTGACTTCCTGAGCGT	TGAAGAAAGAGAGGCATGTGG	271	271	ISBP	this work
**cfp5304 ●**	TAACCATAGGCACACACCGA	GCCCTCACATGATAAGCCAT	185*	185*	ISBP-derived SNP	**this work**
cfp5306	TTCTGTCAACGGCTTGTCTG	TCAAAAACGCTCTTATATTATGGG	124	124	ISBP	this work
**cfp5308 ●**	TTTATCCCAGCAAAGCATCC	GCGGAGCAAAATGAGTGAAT	-	233	ISBP	**this work**
cfp5310	TTGAGGGACAAGGTTTTTCG	GCCTACCCTTCAATTTCGTG	119	119	ISBP	this work
**cfp5311**	ACATCAACCGCGTTGTCATA	GAACCCAGCAAATCCTCTGA	237*	237*	ISBP-derived SNP	**this work**
**cfp5313**	GATCAGGGGGACCAATTTTT	CGGTTACAGAACCGGGACTA	241*	241*	ISBP-derived SNP	**this work**
cfp5314	TGTGACACAAACCGGGACTA	TGCGTGCTTTGGTAAGTGTC	286	286	ISBP	this work
cfp5315	TTCTTTCTTGGGGTTTCCCT	GTTTGGGCGACGAGTAATGT	116	116	ISBP	this work
**cfp5316**	AACAACTCCCCCTCGAGTCT	AGGGGCGAATCTGAGTAGGT	-	130	ISBP	**this work**
**cfp5318**	GCCTGCTCAATTTCTTGGAG	CGCCCGTACATATGAGCTTT	171*	171*	ISBP-derived SNP	**this work**
**cfp5319**	CAACATCACTGCCTGGAATG	AACATGCACACCCACATCTC	285*	285*	ISBP-derived SNP	**this work**
**cfp5320**	AGCCGGCATATCATCTTGAG	GATAATTCTAAGACAAGAATTTTGGAA	-	293	ISBP	**this work**
cfp5324	TAAGTTGCAGGATGGCACAA	GAGGGGCCTTATAAACCCAA	259	259	ISBP	this work
cfp5328	TTATGTGCGGGTTCAACGTA	CGTTTTCTGTAGCCAACTACACC	225	225	ISBP	this work
cfp5331	GCGTTTTCCGTCTGTTTACC	GGCGGACTAGCAACTCAAAC	144	144	ISBP	this work
cfp5335	CGACGCATGGCTAGACTACA	TGATCTAAACGCTCGCTCTT	156	156	ISBP	this work
cfp5338	GAATCAAGGCAACCCTTCAA	GGACGAAGGGAGTACGTGTT	172	172	ISBP	this work
cfp5341	GTGGTTCGTAGATGGCAGGT	CCGCTTAGCGATCTACAAGG	268	268	ISBP	this work
cfp5342	GGGTGCATGTATCAGGCTTT	CGCTGAGATGAGATGAACCA	290	290	ISBP	this work
cfp5352	TCATGAACCGGGACTAAAGG	CGCTGTGGGAGTAAGAGGAG	135	135	ISBP	this work
cfp5354	CGGACTACCAACGGAGACAT	GCGAGTTCCAAGTGCATGAT	254	254	ISBP	this work
**cfp5355 ●**	CAGGAAGCATCGAACACTGA	AAAATCGCCTATTTACCCCC	243*	243*	ISBP-derived SNP	**this work**
**cfp5358 ●**	CGGATTGACATATGACGCTG	TCCCTCCGTCCTAAATTTTTATT	237*	237*	ISBP-derived SNP	**this work**
**CoA ●**	TATGCACATGAATTGAAACACAC	ACAATTTCTGATTCAAGGATACG	-	800	EST	**Mago R., personal communication**
**csSr2RK ●**	AACCAACAAGGTACACATTCTCTG	TATTCTACCAATGGTTGCAACTTC	300	-	EST	**MagoR., personal communication**
**D10F-C5 ●**	ACTGAGTTGGCCCATCGAAC	ACCGAACGGAATCTTCCAGA	200	-	EST	**Mago R., personal communication**
**DOX_1 ●**	AGGAGGCCCCAGAATGATTT	GCTCCGTCACTCTCCTTCGT	700	1100	EST	**Mago R., personal communication**
**ger9-csSr2 ●**	CAAGGGTTGCTAGGATTGGAAAAC	AGATAACTCTTATGATCTTACATTTTTCTG	350	-	PCR-CAPS	**[96]**
NBS	AGTTCACCGCAGTACTGTAGG	CCATGATCACGTCGGTTTTAGG	509	509	EST	**Mago R., personal communication**
**nw1821 ●**	-------------------------	------------------------------	259	252	SSR	**Syngenta**
**Sr2 CAPS ●**	AGTTTAGTCCATCAATAATTTGTG	CTGTGGCCATGTATGGATATACTT	850	-	PCR-CAPS	**Mago R., personal communication**
**stm538acag**	ATCATGTCGATCTCCTTGACG	ACACACACACACACAGAGAGAG	85	80	stm	**[50]**
**stm598tcac**	GTTGCTTTAGGGGAAAAGCC	TCTCTCTCTCTCTCACACACAC	75	70	stm	**[50]**
sts69	AAGGAACGCTGATCCAAATG	TATGACCTCGCTCATTGTCG	216	216	sts	[49]
sts92	CGACGACGTAGATCCAGATG	TGCTCATGATCGTCATCTCC	322	322	sts	[49]
**wmm1104**	TTCTTGAGGATACCGCGTTT	GCATCTGGCGTCTTTGTTCT	184	175	SSR	**Sourdille P., personal**
