# Supplementary material for: Characterization and Use in Wheat Breeding of Leaf Rust Resistance Genes from Durable Varieties
Source: Biology (Basel). 2021 Nov 12;10(11):1168. doi: 10.3390/biology10111168 (PMC8615195; doi:10.3390/biology10111168)
Supplement: Supplementary file 1 [file biology-10-01168-s001.zip › Table S2 - Markers LrcSV2-interval.pdf]

Table S2: Primer sequences of the designed microsatellites within the gpw4388-FSs34 *LrcSV2* interval. The length (in bp) of the amplified product in Sinvalocho (SV) and Purplestraw (P) is indicated. Polymorphic markers are depicted in bold.

Marker	*Forward primer*	*Reverse primer*	SV	P
**FSs88**	TGATTTCCCTTTCTCCGATG	ACGCAACATCTCCAGTGCTT	**230**	**232**
FSs89	GCTTGGATTTGGGAGTCGT	AGCCGGAGAAGAAGTCCTG	230	230
FSs90	CGCACGTTCTTAATCCAACA	TCCCAGGAGAAAACAATGGA	240	240
**FSs91**	CACAGCCACATCATCTTTCC	ATGACCGTATTATTGCGCCT	**225**	**210**
FSs92	TGATGTGCTGCTTCTGCTTC	AACTGCGTGCTTTTCTCTGC	150	150
FSs93	CCACACATGCCGATGATAAA	GTCAGCAATCAATCCCCAAC	245	245
FSs94	CGAACCAAACCAAACCAAAC	GAAGAGAAGGGAGGGAATGC	260	260
**FSs95**	AGCATTGGGGATGTCTGTTC	CCCATCTATGAATTGGCGAC	**160**	**170**
FSs97	ACGCCATCGACTAATCACCT	TCCGTTACCCTTCACAAAGC	290	290
FSs98	GTTCATGCTAACGTACATCCAA	GACAACCTCTGCTTCCCTCT	225	225
**FSs99**	GGCCTAAAATCCCCAATTTC	AATTTGTGTGTGTGTCTCTCTCTGT	**250**	**240**
**FSs101**	TAGGAGGCGAGGGTAACAGA	ATTGATTTGCGGGACAGAAC	**260**	**245**
**FSs103**	CTCGCAGAACAAGGAGAAGC	AGCGGACATAAGGCGTACAG	**232**	**228**
FSs104	CTTCCCAACACTTTCCAAGG	CAGGTAATAGGGATGCCCAG	140	140
**FSs105**	GTTCCGTGGCAAGCTATTG	CACCGATAAAGGCTCCAAGA	**210**	**223**
**FSs106**	TCCCGCATATACACAGTTGC	GTTCCCGGTGTTTCTCTCAA	**224**	**218**
FSs107	CGGTCTAATGGAACACATTTCA	TCTTCCTCCTCCTCCTCCTC	190	190
**FSs109**	ACTACTGCTGGGTTCATGCC	CGGAGGATTGACCACGTT	**220**	**215**
FSs111	AGTTCCCGTGTGATGGATGT	GGTGCAACGCTCTTGGTC	245	245
**FSs122**	ACTTGCATCCATGATAGGGC	ACACGAGAGAGCGAGAGAGC	**185**	**180**
**FSs124**	AGGCAAGCTGACTGAAAAGC	TGCTGTTGCTGTTGTTGTTG	**260**	**273**
**FSs127**	TCGATGACTTAACCGACGTG	CCTCTGATGCCTCTTCTCCA	**207**	**205**
**FSs128**	GAGGGGCTCTACATCAACCA	CCCAACAAACACACCACAAA	**210**	**215**
FSs129	ACGGCCAGTAGTGCTCAAAT	CTAATTGGGGATGATGGGTG	175	175
FSs130	CCCACACCACATGATTTC	TTCACCAGAAGTACAAAGCAC	222	222
**FSs131**	GTTGGATGGCACAACACATC	TGCACGCCCAATAGTTAAGA	**235**	**250**
**FSs132**	TGAGGGCTAGAGAGCCGATA	ATGCTTTCCTCGTGCTGATT	**180**	**170**
FSs133	TCATCACTTCCATGTGCGAT	CAAACCCTAGAGAAGCAGCG	247	247
**FSs134**	CTGGTCGCCTGTTATTCCTT	TGAATGTCAAAATGGCTGGA	**135**	**170**
**FSs135**	GCCTCTCTGCTTTGTCGC	GCAAATAGCTCACATCTTCCAG	**230**	**225**
**FSs136**	TGTTGATTTGGTTGCTTTCC	GCTGACAAGGATGCAGAGAA	**250**	**270**
**FSs147**	ACGCACACACCACACACAG	GCTGCTCATGTACTGCTGCT	**190**	**205**
**FSs148**	CCATTGCCTCATCTCCATTT	CACCCACTTAATAGCCACGG	**235**	**225**
FSs149	TGGACAGCCACCTCATCATA	TGGGAAGGATTGTTTTCGTC	210	210
**FSs150**	CCTATTTCGACACCCTGCC	GCAAAGCTCGTGGTACATCA	**220**	**218**
